# SNPSelect: A scalable and flexible targeted sequence-based genotyping solution

**DOI:** 10.1371/journal.pone.0205577

**Published:** 2018-10-12

**Authors:** René C. J. Hogers, Marjo de Ruiter, Koen H. J. Huvenaars, Hein van der Poel, Antoine Janssen, Michiel J. T. van Eijk, Nathalie J. van Orsouw

**Affiliations:** Keygene NV, Wageningen, Gelderland, The Netherlands; National Institute of Plant Genome Research, INDIA

## Abstract

In plant breeding the use of molecular markers has resulted in tremendous improvement of the speed with which new crop varieties are introduced into the market. Single Nucleotide Polymorphism (SNP) genotyping is routinely used for association studies, Linkage Disequilibrium (LD) and Quantitative Trait Locus (QTL) mapping studies, marker-assisted backcrosses and validation of large numbers of novel SNPs. Here we present the KeyGene SNPSelect technology, a scalable and flexible multiplexed, targeted sequence-based, genotyping solution. The multiplex composition of SNPSelect assays can be easily changed between experiments by adding or removing loci, demonstrating their content flexibility. To demonstrate this versatility, we first designed a 1,056-plex maize assay and genotyped a total of 374 samples originating from an F2 and a Recombinant Inbred Line (RIL) population and a maize germplasm collection. Next, subsets of the most informative SNP loci were assembled in 384-plex and 768-plex assays for further genotyping. Indeed, selection of the most informative SNPs allows cost-efficient yet highly informative genotyping in a custom-made fashion, with average call rates between 88.1% (1,056-plex assay) and 99.4% (384-plex assay), and average reproducibility rates between duplicate samples ranging from 98.2% (1056-plex assay) to 99.9% (384-plex assay). The SNPSelect workflow can be completed from a DNA sample to a genotype dataset in less than three days. We propose SNPSelect as an attractive and competitive genotyping solution to meet the targeted genotyping needs in fields such as plant breeding.

## Introduction

State-of-the-art genotyping solutions have greatly accelerated the speed and efficiency of plant breeding in fields such as cultivar identification, hybrid seed purity testing, (gene) association studies or marker-assisted selection (MAS) [1–4]. Custom-made marker panels are typically used to subject seed lots to quality assurance and quality control procedures in a timely and efficient fashion. For MAS, plants are selected based on genotyping long before the trait of interest is expressed, thereby saving time, labor, space and costs in bringing new varieties to the market. These cost savings are significant, especially when plants can be selected at the seedling stage, particularly for traits such as yield and others that are only visible at the time of crop harvesting.

Maize is widely cultivated throughout the world, with its production increasing yearly [5]. Maize is grown for a variety of purposes and (industrial) products such as food grain, fodder for animals, sweet corn, corn flour, oil, starch, alcoholic beverages, food sweeteners and cosmetics. Improvement of maize through breeding has introduced increased diversity, improved yields and yield stability.

Approximately 80 percent of the maize genome is derived from highly repetitive sequences, interspersed with single-copy, gene-rich regions [6]. The high proportion of repetitive sequences greatly hampers the development of trait-related genetic markers, because conversion of polymorphisms into robust assays may be cumbersome. Hence, efforts to develop genetic markers in maize are directed to targeting the single-copy, gene-rich regions in the genome.

The development of Next Generation Sequencing (NGS) has fueled the discovery of large numbers of Single Nucleotide Polymorphism (SNP) markers and enabled high-resolution, sequence-based genotyping. To date, numerous NGS-based polymorphism discovery and genotyping workflows have been described, which are either based on whole genome (re-)sequencing or involve a form of genome complexity reduction or target enrichment. For example, the Complexity Reduction of Polymorphic Sequences (CRoPS) [7], Restriction-associated DNA (RAD) [8], Genotyping by Sequencing [9] and Sequence-Based Genotyping (SBG) [10, 11] methods target random fractions of the genome in a highly reproducible manner. By contrast, exome sequencing [12], Thermo Fisher’s AmpliSeq [13] and Agilent’s SureSelect [14] target selected loci for comparative re-sequencing. Target enrichment using these methods is accomplished via microarray hybridization, multiplexed PCR and oligonucleotide capture probes, respectively. Libraries of samples processed using these methods are typically sequenced with short-read NGS platforms. Sample barcoding with NGS platform-specific, but assay-agnostic, adaptors or amplification primers, have made it possible to sequence thousands of samples simultaneously with complexity reduction and target enrichment methods. Incremental increases in the output levels of NGS platforms enable screening larger numbers of samples per run and drive further reduction of the costs per sample. Ideally, NGS genotyping workflows are scalable and flexible to take full advantage of these opportunities.

For routine detection of selected SNP marker sets, a wide range of genotyping methods are available. When working with small numbers of markers, monoplex assays such as KASP [15] and rhPCR [16] are very cost effective, particularly when the workflow is automated. However, when hundreds of markers are screened per sample, a tipping point will be reached where multiplexed assays are more cost-effective, even if not all markers are informative in all analyzed samples.

DNA chips [17] and microarrays [18] have been used for hybridization-based multiplexed genotyping purposes. However, a limitation of DNA chips and microarrays is that development- and optimization cycles are costly and time-consuming. Other multiplexed targeted genotyping methods such as SNPWave [19], SNPlex [20], MLPA [21], molecular inversion probes [22] and GoldenGate [23] assays rely on (extension and) ligation of locus- or allele-specific oligonucleotides, followed by amplification with generic primers and detection of amplicons based on length differences or by microarray hybridization. Although powerful because of their uniform workflows irrespective of species and marker content, none of these multiplexed targeted genotyping methods is NGS-based. In addition, because of size-based detection of the amplicons, SNPWave, SNPlex and MLPA assays do not scale to thousands or markers and assay content cannot always be changed between experiments without additional performance optimization.

Altogether, the ideal multiplexed SNP genotyping workflow is highly robust, NGS-based, easily scalable from hundreds to thousands of markers and provides the flexibility to change assay content at will without loss of performance, comparable to the NGS-based target enrichment methods mentioned above.

**Fig 1:**
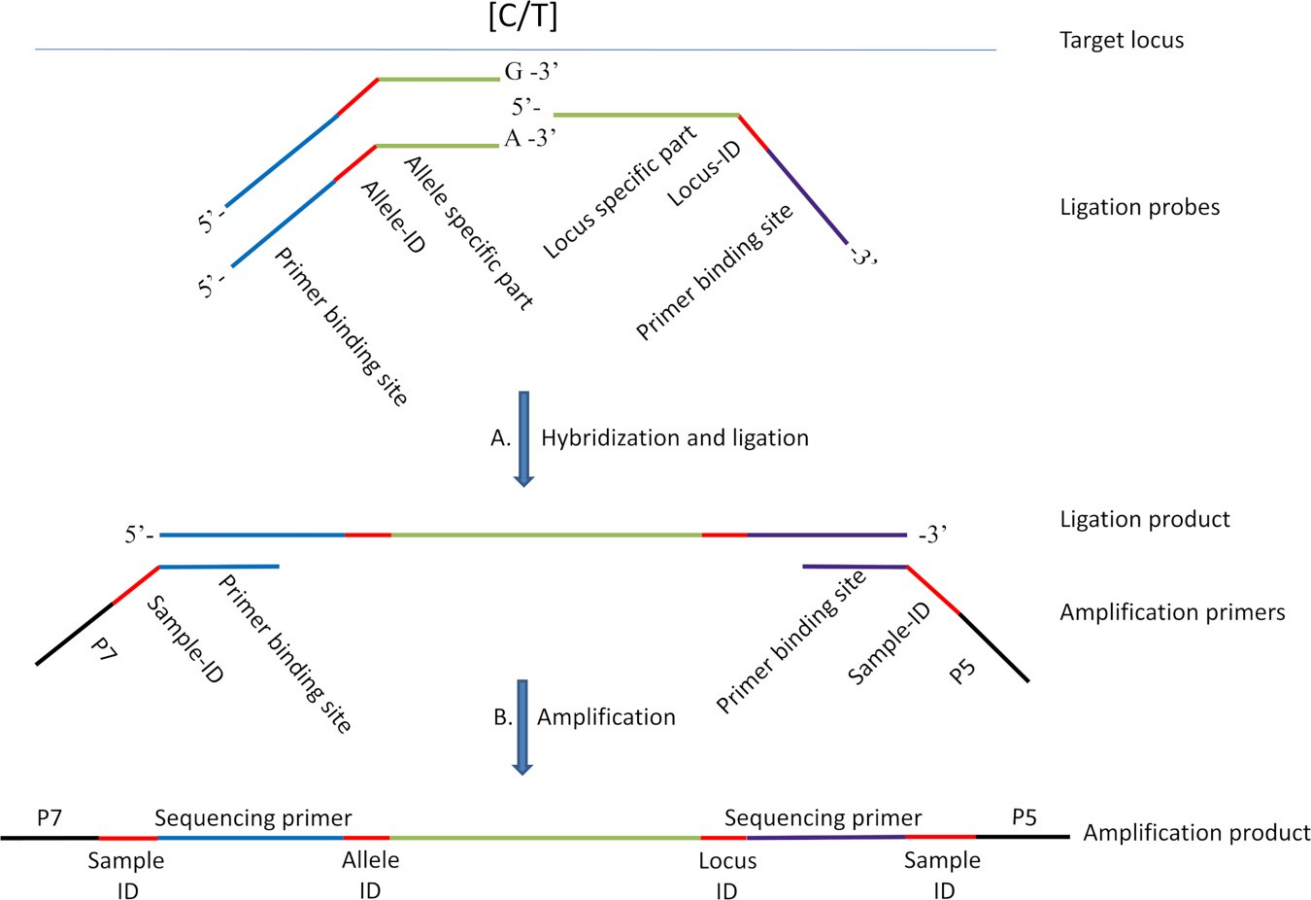
KeyGene SNPSelect technology outline. The KeyGene SNPSelect technology is based on oligo ligation followed by PCR amplification and subsequent sequencing on a NGS platform. Ligation probes contain target specific sequences and barcodes to discriminate loci and corresponding alleles. Amplification primers add barcodes to assign each sequence read obtained to a sample.

Here we present KeyGene SNPSelect, a targeted genotyping solution, which combines the multiplexing capabilities of oligonucleotide-ligation assays with the scalability, flexibility and accuracy of NGS for detection of selected SNP loci. SNPSelect is based on multiplexed ligation oligonucleotide probes containing allele- and locus-specific identification barcodes, followed by multiplexed PCR amplification with generic primers containing sample- and plate-specific barcodes (Fig 1). The amplicons are subsequently pooled, purified and sequenced on a short-read NGS platform (Illumina). We developed a 1,056-plex SNP assay for maize, and present genotyping results from samples of an F2 and a Recombinant Inbred Line (RIL) population and maize germplasm. To demonstrate SNPSelect’s flexibility, we further present genotyping results from subsets of 384 and 768 informative SNP loci selected from the 1,056-plex assay. The overall results demonstrate call rates up to 99.4% and concordance rates up to 99.9% within and between the SNPSelect assays. We propose SNPSelect as a scalable and flexible multiplexed SNP marker genotyping solution for crops and other species.

## Material and methods

### DNA samples

Total genomic DNA was isolated from leaf material using a modified CTAB procedure [24].

**F2 population from a crossing of maize lines PH207 and CO125:** leaf material of an F2 population including the parental lines was kindly provided by KWS SAAT AG (Einbeck, Germany). Genomic DNA from 184 samples of the F2 population and both parental lines was used in the genotyping experiments.

**RIL population:** seeds of the Main Set of IBM RILs and their corresponding parents (B73 and Mo17) were obtained from the Maize Genetics Cooperation Stock Center (Urbana, Illinois, United States of America), grown in the greenhouse, and subsequently leaf material of young plants was harvested. Genomic DNA from 78 RILs and the parental line B73 was used in the genotyping experiments.

**Germplasm:** seeds of germplasm samples were obtained from the U.S. National Plant Germplasm System (Beltsville, Maryland, United States of America), grown in the greenhouse, and subsequently leaf material of young plants was harvested. Genomic DNA from 53 germplasm lines was used in the genotyping experiments.

An overview of all samples including sample names is provided in S9 Table.

### SNPSelect technology outline

SNPSelect is based on the oligonucleotide ligation assay [25]. For each bi-allelic SNP, three oligonucleotide probes are designed: two allele-specific probes that target the respective SNP alleles, whereas the third (common) probe targets the locus immediately adjacent to the SNP site (Fig 1). In addition to their target-specific sequences, each SNPSelect probe contains a specific locus- or allele-barcode sequence and an amplification primer-binding sequence. The locus- and allele-specific probes hybridize to the target locus and allele-specific ligation takes place. Primers specific for the amplification primer-binding sites contain sample-specific barcode sequences and either P5 or P7 sequences for Illumina sequencing. Proprietary combinatorial sequence barcodes are used to assign sequence reads to the individual samples. After amplification, the products are ready for sequencing.

### SNPSelect assay design

SNPs from the first set of 1,536 loci selected by Rousselle and colleagues [26] for their application in Essential Derived Variety (EDV) analysis in European and American maize varieties were used as input for the research presented. SNPSelect probes were designed using KeyGene’s proprietary ProbeDesigner software. Probes for 1,056 loci were selected and oligonucleotides were ordered at 10 μM concentration from Integrated DNA Technologies BVBA (Leuven, Belgium). Each allele-specific probe contains a generic sequence allowing amplification of all alleles using a single amplification primer, a 4-nucleotide identifier sequence (xxxx, S1 Table) to differentiate the alleles, and an allele-specific target sequence ranging in size between 20 and 29 bases (for simplicity denoted as N_20_): 5’-TGGAGTTCAGACGTGTGCTCTTCCGATCTxxxx N_20_-3’. Each locus-specific probe contains a locus-specific sequence ranging in size between 20 and 28 bases (for simplicity denoted as N_20_), an 8-nucleotide identifier sequence (yyyyyyyy, S1 Table) to differentiate the loci, and a generic sequence allowing amplification of all loci using a single amplification primer: 5’- N_20_yyyyyyyyAGATCGGAAGAGCGTCGTGTAGGGAAAGAGT-3’. An overview of the probe sequences designed for the SNP loci used in the genotyping experiments is provided in S10 Table.

### SNPSelect assay preparation

For the locus-specific probes, 10 μl of each locus-specific probe was pooled. A total of 60 μl of the pooled probes was phosphorylated in a 100 μl final volume of 1x PNK buffer using 20 U T4 PNK and 20 μM ATP (both from Thermo Fisher Scientific, Waltham, Massachusetts, United States of America). The mixture was incubated for 60 minutes at 37°C, followed by 10 minutes at 70°C.

The allele-specific probe pool was generated by adding 5 μl of each allele-specific probe. Sixty microliters of the allele-specific probe pool was combined with 100 μl of the phosphorylated locus-specific probe pool, and further diluted to 780 μl (384-plex), 3,900 μl (768-plex) or 2,840 μl (1,056-plex) using Milli-Q (MQ). The diluted final probe mixtures were stored at -20°C until further use.

### Sequencing library preparation

Libraries for Illumina dual-indexed single-read sequencing were prepared as follows: 150–200 ng genomic DNA was mixed with 1 μl of the diluted final probe mixture and 4 U of *Taq* DNA Ligase (New England Biolabs, Ipswich, Massachusetts, United States of America) in a total volume of 10 μl of 1x *Taq* DNA Ligation buffer, denatured for 90 seconds at 94°C, after which hybridization and ligation was performed during a cool down to 60°C and an overnight incubation at 60°C. Ligation reactions were diluted 4-fold, from which 10 μl was amplified in a 40 μl reaction using Phusion Hot Start Flex 2x Master Mix (New England Biolabs) and 2 pmol of primers containing the Illumina P5 or P7 flow cell oligo nucleotide sequences as well as identifiers and (parts of) the Illumina TruSeq paired-end sequencing primers. Sequences of the amplification primers were as follows: P5-primer 5'-AATGATACGGCGACCACCGAGATCTACACxxxxxxACACTCTTTCCCTACAC GAC-3' and P7-primer 5' CAAGCAGAAGACGGCATACGAGATyyyyyGTGACTGGAGTTCAGACGTGT-3' respectively. In these sequences xxxxxx and yyyyy denote 6-base and 5-base combinatorial sequence barcodes (S2 Table). P5 and P7 amplification primers were ordered from Integrated DNA Technologies BVBA. For each sample a unique combination of identifiers was used. Amplification was performed using a thermal profile that consisted of 30 seconds at 98°C, followed by 29 cycles of 10 seconds at 98°C, 15 seconds at 65°C, and 15 seconds at 72°C. Reactions were held at 72°C for 5 minutes and subsequently maintained at 4°C until further use. From each group of 96 samples, 10 μl of each PCR was pooled and purified using the QIAquick PCR purification kit (Qiagen, Hilden, Germany). Purified products were eluted using 30 μl elution buffer (EB) from the QIAquick PCR purification kit. Selection of the amplified ligation products was performed via size selection using the Pippin Prep (SageScience, Beverly, Maryland, United States of America). A 3% agarose gel cassette was used with a size-selection window of 170–230 bp. Size-fractioned products were collected from the elution well and the elution well was rinsed using 40 μl EB + 0.1% TWEEN, which was combined with the initial eluate. The total eluate was purified using the MinElute Purification kit (Qiagen) and eluted in 15 μl EB. The quality of the library was assessed via analysis on the Agilent Bioanalyzer (Agilent, Santa Clara, California, United States of America) using a High Sensitivity DNA Chip. Concentration of the library was determined with Qubit fluorometric quantitation (Thermo Fisher Scientific) using the Qubit dsDNA HS Assay Kit.

### Sequencing

Dual–Indexed Single-Read sequencing (126nt) was performed using the HiSeq2500 (Illumina, San Diego, California, United States of America). Clusters for each library were generated on a HiSeq flow cell v4 using a TruSeq Single-End Cluster Kit v4, according to the manufacturer’s instructions. Upon completion of the sequencing run using HiSeq2500 SBS v4 chemistry, image analysis, error estimation and base calling were performed using the Illumina Pipeline (HCS 2.2.68 / RTA v1.18.66.3). Bcl2fastq v2.17.1.14 was used to sort and pool sequences into single (zipped) .fastq files per lane / per sample. All sequence files were deposited in the NCBI Sequence Read Archive (SRA) with accession number SRP157886.

### Processing de-multiplexed data and SNP genotyping

De-multiplexed sequencing files were processed using customized scripts implemented in a Galaxy environment [27]. The scripts assign each sample read to a locus-allele combination present in the assay. Read assignment was based on the nucleotide distance in the sequence reads between the identifiers used for the loci and alleles in the assay. Results were exported as a tab-delimited file containing counts for the alleles in each sample/locus combination. Next, the tab-delimited data file containing the counts for all sample, locus and allele combinations was imported in proprietary software in which single character genotype calls (A, B or H) were automatically assigned to a sample-locus combination based on ratio scores. The software defines the three genotype classes based on the calculated ratio between the read counts for allele-1 and allele-2, for each individual sample of a specific locus. The genotyping software allows for optional manual adjustment of the boundaries between the genotypic classes.

### Genotype validation

In order to determine the accuracy of the genotypes determined using the SNPSelect technology, reproducibility was assessed by comparing the genotypes obtained for DNA samples that were included multiple times. Further validation was performed by comparison of the genotypes to published data obtained using Illumina MaizeSNP50 arrays [28] to determine the concordance rates. The overlap between the two data sets comprised a total of 29 germplasm lines and 1,037 SNP loci. Finally, the accuracy of genotyping of the same SNP loci included in different assays with varying multiplexing levels was determined. For this, the genotypes obtained from the loci present in the 384-plex, 768-plex and the 1056-plex were compared.

### Linkage mapping and map comparisons

Genetic maps for the F2 and RIL populations were calculated using the CarteBlanche software package [29]. First, genotype scores from the .loc file were filtered for informative high-quality markers. Filtering removed markers with >25% missing data-points per marker, non-segregating markers, and markers with extreme segregation distortion (P < 0.000001 based on chi-squared test, i.e. observed segregation vs expected segregation). As markers scored in RIL populations are in general more difficult to map, a more stringent threshold for missing data was used, i.e. only markers with fewer than 5% missing data-points were used. Next, these high-quality markers were imported in CarteBlanche, which is a genetic mapping software program allowing estimation of linkage groups, determination of the most likely map orders using various mapping algorithms, and various visualization methods and statistics to judge mapping quality.

Genetic maps for the populations were calculated individually and subsequently compared.

## Results

### Development and application of a 1,056-plex maize SNPSelect assay

A 1,056-plex maize SNPSelect assay was designed, developed and applied to a collection of 374 samples from F2 and RIL populations and a germplasm collection. SNPSelect amplicons sequenced on a HiSeq2500 produced a total of 205,270,919 filtered and de-multiplexed reads, with an average of 548,853 reads per sample.

### SNP genotyping using a 1056-plex SNPSelect assay

Sequencing reads for each sample were processed, and the resulting allele frequencies for each locus and sample were exported to a read count file in tab-delimited format (S3, S4 and S5 Tables). SNP alleles are indicated as A, C, G, T, or as + or–to indicate insertions and deletions, respectively.

The read count file containing the sample, locus and allele frequencies was subsequently imported in proprietary scoring software and the genotypes were determined. Allele frequencies are presented in a graph as “raw” frequencies and as ratio scores (S1 and S2 Figs).

Resulting genotypes were exported in the .loc file format. A summary of the genotype calls is shown in S6, S7 and S8 Tables. Call rates varied between 81.2% and 91.6%, depending on the dataset (Table 1). It must be noted that since the SNP loci are identified using the genome sequence of B73 as a reference, it can be expected that the germplasm collection may represent different sequences (i.e. some markers could be absent resulting in a lower call rate).

**Table 1:** Genotype call rates for the maize SNPSelect 1,056-plex assay.

Data set	Samples	Maximum # genotypes (# samples x # SNPs)	Called genotypes	Call rate^a^
F2	190	200,640	183,859	91.6%
RIL	91	96,096	84,236	87.7%
Germplasm	93	98,208	79,747	81.2%
Total	374	394,944	347,842	88.1%

^a^ = % of genotypes called (i.e. excluding U scores).

### Genotype concordance and reproducibility in 1,056-plex dataset

Genotype concordance (i.e. the percentage of identical genotypes between two datasets) was determined by comparing previously published genotypes from 29 germplasm samples [28] with the genotypes of the same samples obtained with 1,037 loci from the 1,056-plex SNPSelect assay. Based on a total of 24,368 available datapoints, the concordance rate was 97.6% (Table 2).

Reproducibility (i.e. the percentage of identical genotypes from duplicated samples within a dataset) was calculated for 4 F2, 12 RIL and 40 germplasm samples and resulted in 99.9%, 98.8% and 97.9% reproducibility rates, respectively, i.e. 98.2% on average based on a total of 56 samples.

**Table 2:** Genotype comparison of maize SNPSelect 1,056-plex assay data to published data.

Genotype	Count	Sum
SNPSelect vs public data set		
A vs A	11166	24069
B vs B	12607	
H vs H	3	
U vs U	293	
A vs U or U vs A	2768	5412
B vs U or U vs B	2641	
H vs U or U vs H	3	
A vs H or H vs A	11	592
B vs H or H vs B	8	
A vs B or B vs A	573	
Total genotype calls (29 samples x 1037 loci)	30073	
Genotype calls present in both data sets	24368	81.0%
Concordant genotype calls	23776	97.6%

### Linkage mapping

To get an impression about the quality of the genotypes, linkage mapping within the F2 and RIL populations was performed. First, genotyping data was filtered for informative high-quality markers. Filtering removed markers with an overrepresentation of a single genotype (A vs H vs B). To achieve this, per marker the fraction of the most occurring genotype score was determined and only markers that contained less than 90% of the same genotype remained. A total of 432 informative high-quality markers were obtained, of which 145 were scored in the RIL population, 287 in the F2 population and 43 were informative in both (Table 3). Hence, these 432 markers represent 389 SNP loci. These markers were used as input to perform linkage mapping using the CarteBlanche software [29]. Linkage maps for the F2 and RIL populations were visualized (S3 and S4 Figs). A total of 328 out of 389 unique markers (84.3%) were mapped in at least one of the two populations. Of these 328 markers, 305 occur on one genetic map and 23 in both. Consistent with reliable genotyping, the vast majority (93.0%) of the high-quality markers that were scored in the F2 population were placed on the linkage map. This fraction was significantly lower (57.9%) for the RIL population, which is not unexpected because there is less overlap between regions containing markers in RILs compared to samples of an F2 population. This means that in order to link markers that are located far apart on the genome with confidence, more in-between markers are needed for the RIL population than for the F2 population. Since both populations have been genotyped with similar density, it can be expected that not all the distant markers of the RIL population can be linked. Mapping RIL markers with less stringency in order to place more markers on the map, yielded a lower quality map. Here we only present stringent mapping, which resulted in a higher quality map. The mapped markers were placed in 14 and 13 linkage groups, with cumulative map lengths of 1,097 and 439 cM for the F2 population and the RIL population, respectively.

**Table 3:** Filtering steps and results per mapping population.

Population	Step	# Markers remaining
F2	Start	1,056
F2	Remove markers with > 25% U-scores	955
F2	Remove markers with overrepresented genotypes (>90% same genotypes)	313
F2	Remove markers with P < 0.000001 (Chi-square for segregation distortion)	287
F2	Final (ready for mapping)	287
RIL	Start	1,056
RIL	Remove markers with > 5% U-scores	569
RIL	Remove markers with overrepresented genotypes (>90% same genotypes)	145
RIL	Remove markers with P < 0.000001 (Chi-square for segregation distortion)	145
RIL	Final (ready for mapping)	145

### Selection of subsets from the 1,056-plex SNPSelect assay

In order to increase the information content of the assay and thereby decreasing the genotyping costs further, the most informative loci were selected from the 1,056-plex SNPSelect assay. To make this selection, first loci containing <25% unknown genotype (U) scores in at least one of the three genotype datasets were selected. Subsequently, from these loci a total of 876 loci were selected.

### Assembly and sequencing of 384-plex and 768-plex SNPSelect assays

A 384-plex and a 768-plex SNPSelect assay was assembled using a subset of the 876 selected loci. The selection of loci for both assays was done as follows. First, for the 384-plex, 342 loci were selected from the total of 552 which segregated in at least two datasets. The remaining 42 loci were segregating in a single dataset. For the 768-plex, these 384-plex loci were supplemented with 193 loci segregating in at least two datasets, 190 loci segregating in a single dataset and one locus which did not segregate in the samples tested, such that a total of 535 loci of the 768-plex segregated in at least two datasets.

**Fig 2:**
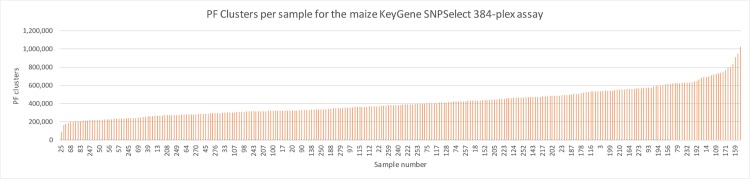
Passing filtering cluster distribution across the 254 samples genotyped. Passing filtering (PF) cluster number distribution across the 254 samples genotyped using the SNPSelect 384-plex assay. Values on the X-axis indicate the sample numbers. The Y-axis shows the PF cluster numbers.

The 384-plex and 768-plex assays were subsequently applied to a collection of 283 samples (including 29 duplicates) consisting of F2, RIL and germplasm samples, of which the majority was also used for genotyping with the 1,056-plex assay. Amplification products were generated and subsequently sequenced on a HiSeq2500. The total number of filtered and de-multiplexed reads generated was 116,971,732 and 118,530,099 for the 384-plex and 768-plex, respectively. Average reads per sample was 413,328 (384-plex) and 418,834 (768-plex). An example of the read distribution across the samples genotyped using the 384-plex assay is shown in Fig 2. Genotype call rates for the 384-plex and 768-plex were 99.4% and 96.5%, respectively.

### Genotype concordance and reproducibility in 384-plex and 768-plex datasets

Concordance rates for the 384 SNP loci present in the 384-plex, 768-plex and 1,056-plex SNPSelect assays were determined. Concordance rates were 97.9% (384-plex vs. 768-plex), 98.0% (384-plex vs. 1,056-plex) and 98.4% (768-plex vs. 1,056-plex) (Table 4).

**Table 4:** Genotype concordance of the 384 SNP loci compared to the 384-plex, 768-plex and 1,056-plex SNPSelect assays.

Data set comparison	# Genotypes in comparison (254 samples x 384 SNPs)	# Called genotypes	% Called genotypes^a^	# Concordant genotypes	% Concordancy
768-plex vs. 1,056-plex	97536	93671	96.0%	92145	98.4%
384-plex vs. 1,056-plex	97536	94717	97.1%	92813	98.0%
384-plex vs. 768-plex	97536	95511	97.9%	93538	97.9%

^a^ = % of genotypes called in both data sets, excluding U scores

Genotype reproducibility in the 384- and 768-plex SNPSelect assays was calculated by determining the percentage of identical genotypes between the genotypes obtained from 29 duplicated samples, and were 99.9% and 99.3% for the 384-plex and 768-plex, respectively.

## Discussion

We have developed SNPSelect and presented results of a 1,056-plex assay applied in 374 maize samples of F2 and RIL populations and a germplasm collection, with call rates between 81.2% and 91.6% and reproducibility rates of samples tested in duplicate of at least 97.9%. SNPSelect genotypes were benchmarked with published Illumina MaizeSNP50 array data for a subset of 1,037 loci scored in 29 germplasm samples with a concordance rate of 97.6%. The majority of the small proportion of discordant results were attributable to a subset of six samples, and this was not investigated further. Furthermore, we demonstrated the versatility of the SNPSelect technology by creating 384-plex and 768-plex assays based on the most informative subsets of markers from the 1,056-plex assay, and presented concordance rates between genotypes obtained using these assays of 97.9% and higher. These results showcase the potential of the SNPSelect technology for routine genotyping of selected SNPs in a highly customizable and easily adjustable multiplexed format without additional protocol optimization in a crop with a complex repetitive genome. The SNPSelect workflow can be completed from genomic DNA to genotype dataset in less than three days when starting with pre-validated assays. SNPSelect combines highly reliable multiplexed ligation-based allele discrimination with the throughput, scalability and cost advantages of short-read NGS platforms. Contrary to sequence-based genotyping methods based on genome complexity reduction methods, all NGS reads produced from SNPSelect assays are expected to contribute to a genotype data point, which lays the basis for obtaining extremely high numbers of genotypes per run at minimal costs. Indeed, with an average redundancy of more than 375 reads per SNP locus per sample, at least 400 samples can be scored for 1,000 SNPs (or equivalent combinations of SNPs and samples) using a single lane of a HiSeq 2500 flow cell. This equals production of 400,000 SNP genotypes per lane or 6.4 million genotypes per run with two flow cells. It is expected that this throughput can be increased further by accepting a lower average redundancy of reads per genotype. In practice, the combination of an easily adjustable SNPSelect assay incorporating our proprietary combinatorial sequence barcoding methods for sample pooling and the availability of a variety of NGS platforms covering a wide range of output levels (such as Illumina’s Miseq, Hiseq and NovaSeq platforms, and others) allow users to tailor SNPSelect to their specific project requirements to optimize sample throughput, turnaround times and/or cost per data point. To date, in addition to maize we have performed SNPSelect in a variety of crops of different genome sizes and complexities including oil palm, carrot, melon, eggplant, radish and lettuce, with multiplexing levels ranging from 192 to 10,000 SNPs per assay using either MiSeq or HiSeq2500 platforms.

A unique feature of SNPSelect assays is the use of locus- and allele-specific identifier sequences in the ligation probes. This enables counting reads representing alleles based on the combination of locus- and allele- specific barcode sequences instead of detecting the actual SNP position in the sequence read. Our data analysis process incorporates this approach and further takes into account the nucleotide distance between the locus- and allele-specific barcodes. In this way, sequence reads obtained from incorrect ligation products are excluded from further processing. These filtered data are subsequently used for (automated) genotype scoring. Our scoring software delivers A, H, B and intermediate C (B or H) or D (A or H) single character genotype calls which can directly be used for downstream applications such as genetic map construction using CarteBlanche. Besides scoring in a fully automated way, the software allows user intervention such as manually setting the boundaries between genotypic classes, which may be advantageous when scoring sample collections with skewed allele frequencies.

Continuous improvements of short-read NGS platforms in terms of throughput, run times and cost per base have sparked development of numerous sequence-based genotyping methods screening either random loci based on genome complexity reduction approaches or targeting specific loci using probes or primers. Random and targeted sequence-based genotyping methods are complementary as both come with specific advantages and limitations such that a “one-fits-all” solution in terms of optimal information density, cost and speed does not apply. For example, specific advantages of genome complexity reduction methods are that no genome sequence information is required and that generic reagents (such as barcoded adaptors) can be deployed across multiple species, which reduces upfront investments costs. Furthermore, genome complexity reduction methods enable both marker discovery and genotyping in a single process. However, downsides of genome complexity methods are that the number of informative markers is difficult to predict and requires fine-tuning per species and that the cost per data point may be higher than for targeted methods as not all sequence reads are informative. By contrast, targeted sequence-based genotyping methods do require prior genome sequence information to enable locus-specific primer- or probe design and upfront investment in these assay reagents. However, advantages are that they can also be used for combined marker discovery and genotyping in a single process and deliberately targeted to known polymorphisms linked to important traits. In addition, the genotyping datasets are consistent for all samples and the cost per marker is often lower than for random methods, provided that (cumulatively over time) a sufficient number of samples are processed to amortize the probe- and primer reagents costs to minimal levels. In this landscape SNPSelect represents a unique technology enabling routine genotyping of hundreds to thousands of known SNPs or small indels per sample in a single reaction vessel based on the proven OLA for allele discrimination. Improvements of SNPSelect are directed at reducing the upfront investment costs of ligation probes and establishing the upper boundaries for multiplexing such that in the future it will be possible to genotype thousands of markers per sample for high-density genetic mapping or genome-wide selection processes in crops at significantly lower costs and with higher fractions of informative markers than fixed content DNA chips.

In conclusion, KeyGene SNPSelect offers a fully customizable, highly flexible, multiplexed SNP genotyping solution leveraging the power and accuracy of NGS-based detection. It provides scalable flexibility in terms of numbers of SNP loci and samples per project, and assay content that can be adjusted between projects to reduce costs. SNPSelect genotyping can find widespread application for (highly) multiplexed genotyping in any organism.

## Supporting information

S1 Fig Raw allele frequency plot for locus PZE-102114559. Each sample is represented as a single dot. The x- and y-axis represent the obtained read counts for the A and B allele, respectively.
(TIF)

S2 Fig Allele frequency ratio score plot for locus PZE-102114559. Each sample is represented as a single dot.
(TIF)

S3 Fig Linkage map obtained using the data from the F2 population samples. (TIF)

S4 Fig Linkage map obtained using the data from the RIL population samples. (TIF)

S1 Table Allele- and locus- barcode nucleotides used in the maize SNPSelect 1,056-plex assay. (XLSX)

S2 Table Combinatorial sequence barcode nucleotides for de-multiplexing SNPSelect sequencing data. (XLSX)

S3 Table Preprocessing results from the de-multiplexed sequencing data of the F2 population. (TXT)

S4 Table Preprocessing results from the de-multiplexed sequencing data of the RIL population. (TXT)

S5 Table Preprocessing results from the de-multiplexed sequencing data of the germplasm samples. (TXT)

S6 Table Genotyping results from the F2 population. (TXT)

S7 Table Genotyping results from the RIL population. (TXT)

S8 Table Genotyping results from the germplasm samples. (TXT)

S9 Table Overview of samples and samples names used in the genotyping experiments. (XLSX)

S10 Table Overview of probe sequences designed for the loci used in the genotyping experiments. (XLSX)

S1 Scripts SNPSelect preprocessing scripts. (ZIP)
