# Supplementary figures and images for: SNPSelect: A scalable and flexible targeted sequence-based genotyping solution
Source: PLoS One. 2018 Oct 12;13(10):e0205577. doi: 10.1371/journal.pone.0205577 (PMC6185863; doi:10.1371/journal.pone.0205577)

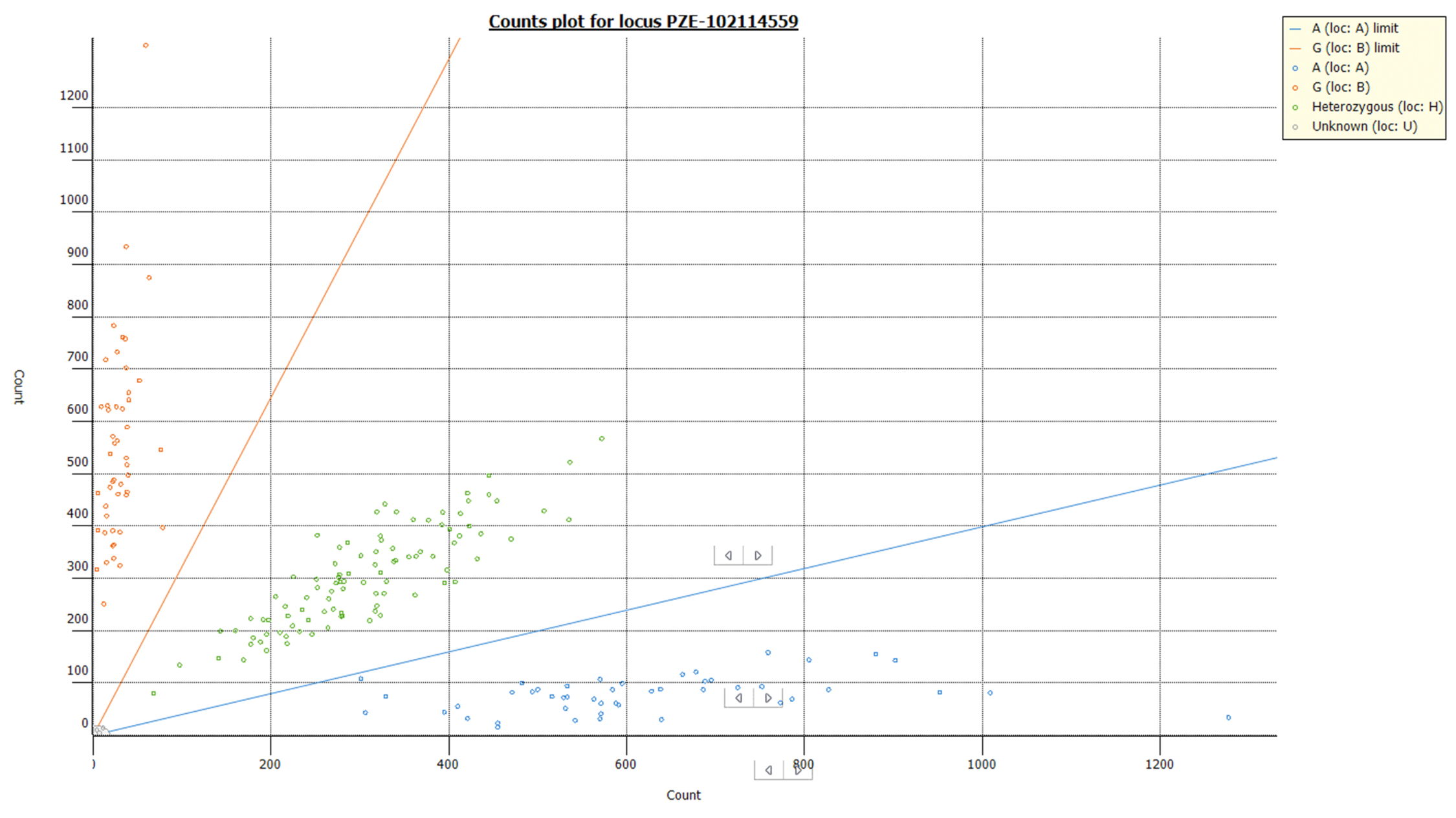

Supplement: S1 Fig — Each sample is represented as a single dot. The x- and y-axis represent the obtained read counts for the A and B allele, respectively. (TIF) [file pone.0205577.s001.tif]

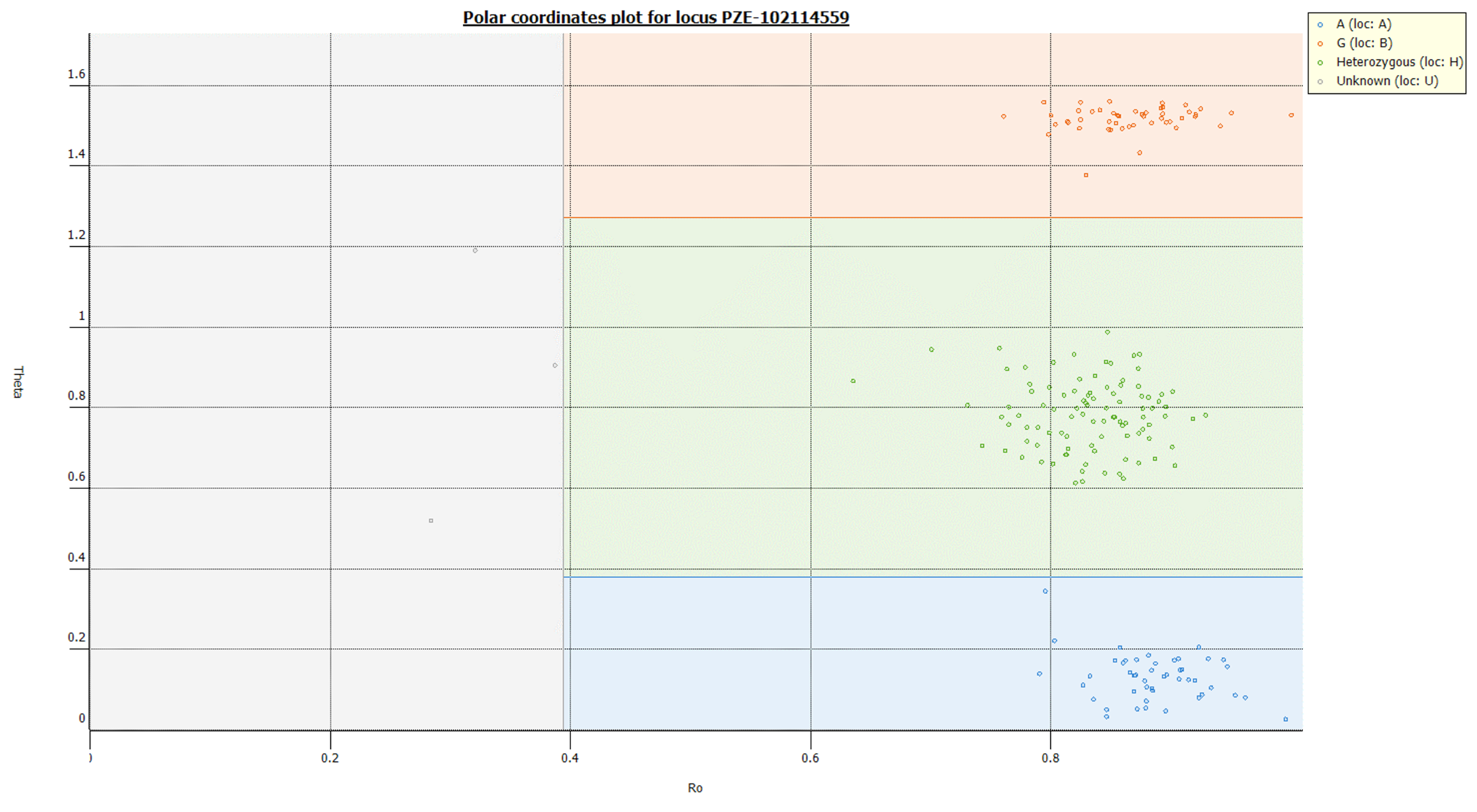

Supplement: S2 Fig — Each sample is represented as a single dot. (TIF) [file pone.0205577.s002.tif]

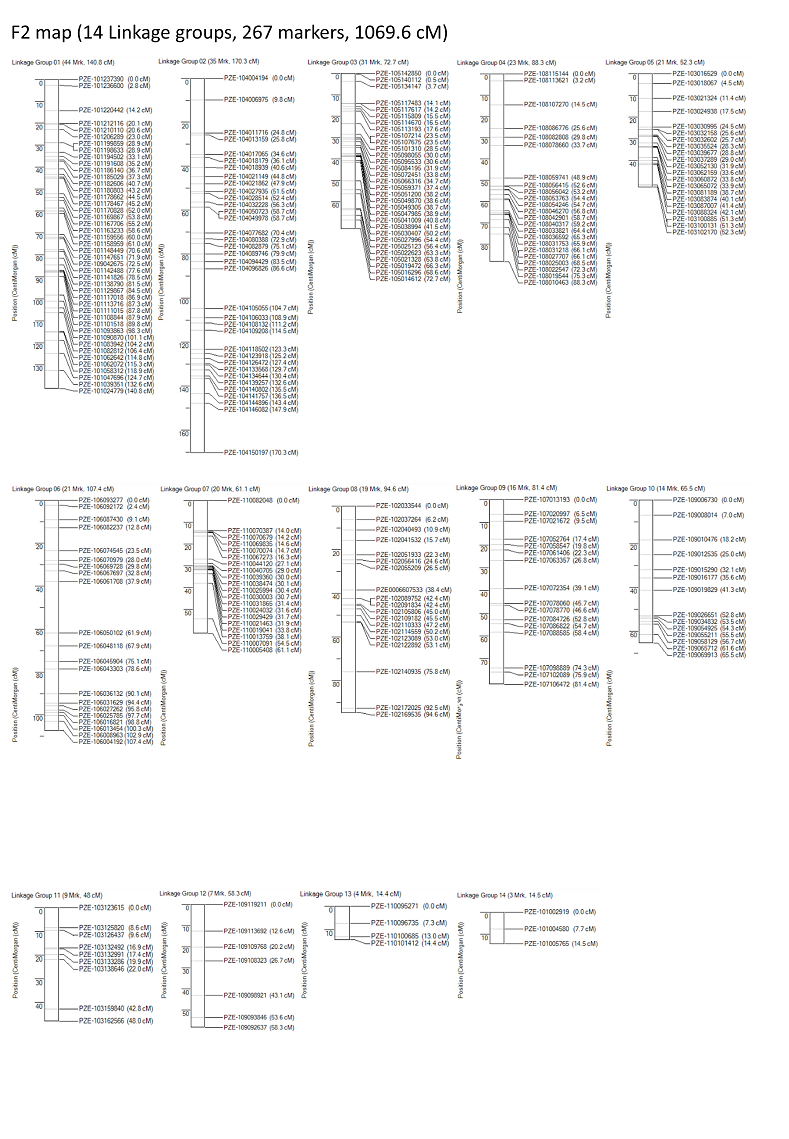

Supplement: S3 Fig — (TIF) [file pone.0205577.s003.tif]

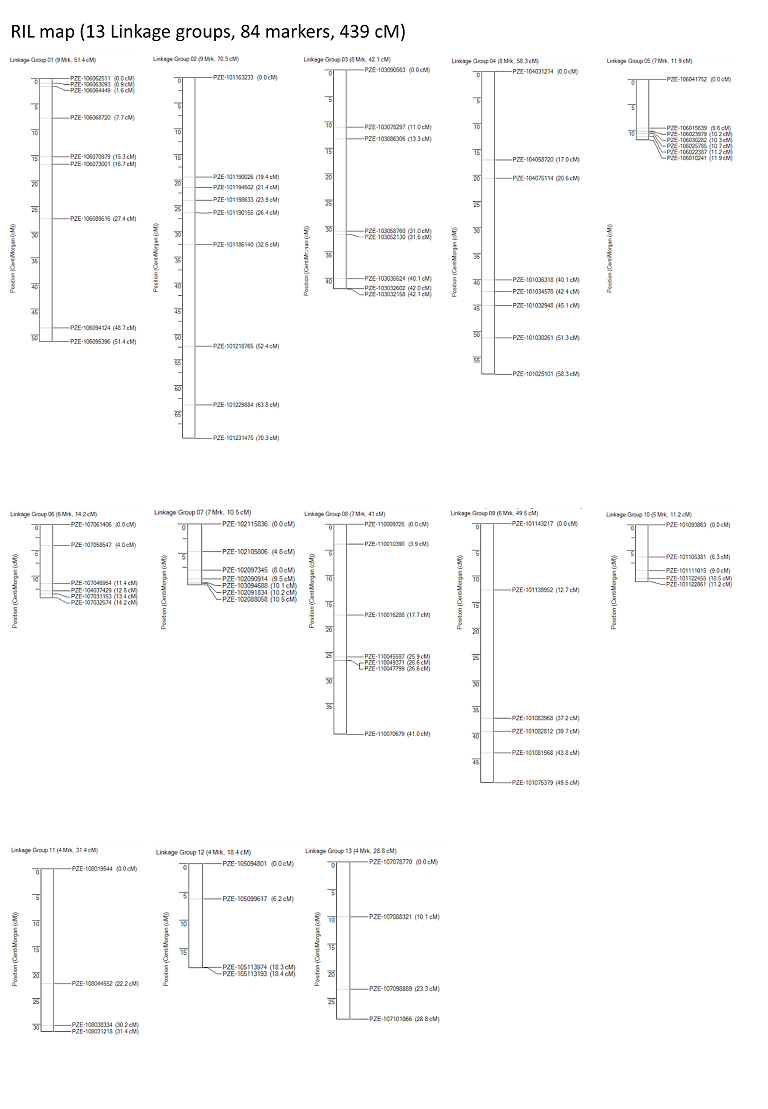

Supplement: S4 Fig — (TIF) [file pone.0205577.s004.tif]
